# Quality of life in chronic musculoskeletal symptomatic Chilean population: secondary analysis of National Health Survey 2009–2010

**DOI:** 10.1186/s12891-020-03261-x

**Published:** 2020-04-21

**Authors:** Maria Jesus Mena-Iturriaga, Manuel Vicente Mauri-Stecca, Phillip S. Sizer, Jaime Leppe

**Affiliations:** 1 Physical Therapy School, Universidad del Desarrollo, Avenida Plaza 680, Las Condes, Santiago, Chile; 2 Advanced Physical Therapy, 1917 Abbott Rd Suite 200, Anchorage, AK 99507 USA; 3 Department of Rehabilitation Sciences, Texas Tech University Health Sciences Center, School of Health Professions, 3601 4th St.; Room 2B138, Lubbock, TX Zip 79430 USA

**Keywords:** Health-related quality of life, SF-12 questionnaire, Population study, Musculoskeletal symptoms

## Abstract

**Background:**

Health-related quality of life (HRQoL) is defined as the patient’s perception of their health status. HRQoL can be modified by illnesses, treatments or social and health policies. Chronic musculoskeletal pain is a modifying factor of HRQoL that leads to lower quality of life, elevated suffering and disability. Knowing HRQoL in subjects reporting chronic musculoskeletal symptoms (cMSS), like pain, discomfort or swollenness lasting more than 3 months, will provide information to health teams and organizations engaged in the Chilean health system. This study aim was to determine the relationship between HRQoL and musculoskeletal symptoms measured in three different Chilean groups: [1] without symptoms; [2] with acute symptoms; and [3] with cMSS.

**Methods:**

A secondary analysis of the 2009–10 Chilean National Health Survey (NHS) was executed to determine the relationship between HRQoL (measured with SF-12) in three MSS groups. The Chilean NHS considered a national, probabilistic, stratified and multistage sample of 5293 participants aged 15 and older; it was representative at the national, urban-rural and regional levels. A multivariate logistic regression model studied the relationship between cMSS and HRQoL, adjusted for age, sex, educational level and residence area as control variables (*p* < 0.05).

**Results:**

Out of 5293 participants in the NHS 2009–10, 5276 subjects were included for analysis. The median age was 46 years (IQR 31–60), 59.4% women, a median of 10 years formal education (IQR 7–12) and an urban residence in 85.2% of the population of the NHS 2009–10. The observed population prevalence of people with cMSS was 42.6% (95% CI 40.4–44.9). Presence of cMSS is a risk factor for low HRQoL, exhibited both in the physical (OR 3.1 95% CI 2.7–3.5) and mental (OR 1.9 95% CI 1.6–2,) HRQoL dimensions, independent of control variables.

**Conclusions:**

Physical and mental HRQoL are affected in people with cMSS, low educational level and advanced age. This is especially seen in women. This information will facilitate assessment and treatment of cMSS as a prevalent and multidimensional health problem.

## Background

Health-related quality of life (HRQoL) can be defined as the patient’s perception of their well-being, health status and length of life. A person’s HRQoL can be modified by factors such as impairments, functional states, perceptions and social opportunities. Such HRQoL can be altered by diseases, injuries, treatments or social and health policies [1–3]. Chronic pain is a modifying factor of HRQoL, impacting individuals in different degrees and leading to suffering, disability, and reduced quality of life [4, 5].

Chronic pain is a pain experience that persists beyond the normal time that is commonly required for tissue healing, or approximately 3 months [6]. Chronic pain is a global health problem present in 19% of adult European population [4], versus the 40% found in Latin America (Colombia 33.9%, Brazil 42.3%), [7, 8] and 34% in medium-to-low income countries [9]. Chronic musculoskeletal pain constitutes the second cause of disease burden both to those who suffer and to the health systems where they are immersed [10].

There is, in this way, the need to assess the musculoskeletal health problem. In Chile, the National Health Survey conducted between 2009 and 2010 (NHS 2009–10) identified MSS through the “COPCORD Questionnaire CCQ-ILAR” adapted version [11, 12]. This questionnaire showed that 37.6% of Chilean population reported the presence of non-traumatic MSS without considering chronic or non chronic symptoms. HRQoL of Chilean population was descriptively reported in the NHS 2009–10 using the “12-Item Short Form Health Survey” (SF-12) in its 2.0 version. The results from SF-12 showed that 17.8% of the population reported experiencing pain during the previous 4 weeks, which interfered “fairly” or “a lot” with their activities.

Nevertheless, the NHS 2009–10 final descriptive analysis did not present any of the following data: a) Without MSS, acute MSS and cMSS (chronic musculoskeletal symptoms) prevalence; b) specific score analysis of SF-12 physical and/or mental domains, as it has been suggested in the literature [13]; and c) the relationship between HRQoL and cMSS at the population level. It is relevant to know the relationship between these constructs, considering that the assessment of HRQoL allows the evaluation to deepen the subjective dimension of pain within a specific population [1], monitor that population’s health [2], and evaluate the effect of social and health policies on that population [3].

The objective of this study was to determine the relationship between HRQoL in terms of physical and mental dimensions within the Chilean population without musculoskeletal symptoms (woMSS), with acute musculoskeletal symptoms (aMSS) and with cMSS taken from a secondary analysis of the NHS 2009–10.

## Methods

### Processes and participants

A secondary analysis of the Chilean NHS 2009–10 database was carried out. The former primary study was conducted in households with a national, probabilistic, stratified and multistage sample of 5293 participants aged 15-year-old and older. It was carried out between October 2009 and September 2010. The sample was representative at the national, urban-rural and regional levels, and it was calculated with 20% relative error for national prevalence estimation of over 4% [12]. In the present secondary analysis, the entire sample was considered (census sampling) in order to respect the regional and national representativeness of the original study (NHS 2009–10). The MSS and HRQoL information from the NHS 2009–10 database were measured through the CCQ-ILAR and SF-12, respectively.

### Health related quality of life (HRQoL)

The SF-12 questionnaire v2.0 is a compact version of the SF-36 Health Questionnaire used in the NHS 2009–10, consisting of 12 questions that are grouped into 8 dimensions (physical functioning, functioning of the physical role, body pain, general health, vitality, social functioning, emotional functioning and mental health). The dimensions are gathered in two domains or indexes (physical health and mental health). Each index includes a score ranging from 0 to 100, where higher scores indicate better perceived health status. This questionnaire exhibits a relative validity (validity relative to summary measures and scales based on the SF-36) that ranges between 0.43 and 0.93 (median = 0.67) [13]. To calculate the HRQoL scores, both in terms of physical composite scores (PCS) and mental composite scores (MCS), the questionnaire questions were graded and normalized using the Software QualityMetrics Health Outcomes® Scoring Software 5.0 (license QM044465, Universidad del Desarrollo). The scoring system is a normalized score, with a scale from 0 to 100, being 100 a better HRQoL condition. This analysis delivered a PCS and MCS for each individual and further categorized the scores by standard deviations below the general population at “well below” (> 1 standard deviation), “below” (between < 1 and > 0.5 standard deviation) and “equal or better” (< 0.5 standard deviations) PCS or MCS in relation to the general population [13]. Subsequently, categorization was deduced by standard deviations of HRQoL in: “low HRQoL” for subjects classified “well below” or “below”, and “high HRQoL” for subjects classified as “equal or better”, in both dimensions (PCS and MCS).

### Musculoskeletal symptoms

The COPCORD Questionnaire CCQ-ILAR adapted and validated in Spanish was used in the NHS 2009–10 with a 76% specificity, 92% sensitivity and 0.8 internal validity measured with Cronbach’s alpha [11, 12]. Based on the question: “Have you had any pain, discomfort or swollenness, not caused by a trauma, in the last seven days?”, the questionnaire considered the following basic indicators: (a) MSS1-musculoskeletal symptoms of non-traumatic origin in the last 7 days, independent of the intensity of pain; and (b) MSS2-musculoskeletal symptoms of non-traumatic origin in the last 7 days with intensity greater than or equal to 4 (of a maximum of 10) on the verbal scale of pain. From variable MMS1, the variables were defined as: acute MSS (aMSS) when the presence of MMS1 was less than 90 days; Chronic MSS (cMSS) when the presence of MSS1 was 90 days or more [6]; and without MSS (woMMS) in the absence of reported MSS1.

## Data analysis

The variable “age in years” was described using median scores (interquartile range), due to a nonparametric distribution. The PCS and MCS scores of HRQoL were presented on average (95% CI). Sex, area of ​​residence, age range, educational level, without MSS, aMSS, cMSS, and the dichotomized HRQoL variables were presented in absolute and relative frequencies. Expansion factors (reported in the NHS) and 95%CIs were considered for prevalence calculations. A comparison of scores obtained in the HRQoL (in their PCS and MCS indices) in subjects without MSS, with aMSS and with cMSS, according to the control variables: sex, age in range, educational level and area of residence, was performed. A correlation between both the PCS and MCS indexes of HRQoL with both age and years of study was performed using the Spearman Rho correlation coefficient. Univariate logistic regression was performed to explain low HRQoL in its physical and mental dimensions, independently considering the cMSS variables (compared to the non-presence of cMSS), female sex (compared to male), age in the age range (compared to age of 15–24 years), educational level (compared to high educational level) and rural residence (compared to urban).

In a multivariate logistic regression model, the presence of cMSS was considered as exposure to low HRQoL in its physical and mental dimensions, adjusted to the aforementioned control variables.

Missing data weren’t considered in the analysis. For HRQoL variable, 17 subjects who did not present data on HRQoL were excluded from the analysis. For all analyses, the statistical package STATA 15.0 was used, considering a level of significance of 5%.

## Results

Out of 5293 participants in the NHS 2009–10, 17 subjects who did not present data on HRQoL were excluded, leaving a total of 5276 subjects for analysis. We observed a median age of 46 years (IQR 31–60), 59.4% women, a median of 10 years of study (IQR 7–12) and an urban residence in 85.2% of the population of the NHS 2009–10. The subjects reported in 41.2% the presence of MSS independent of its intensity, and 37.6% of MSS with intensity greater than or equal to 4 (Table 1).

**Table 1:** Sociodemographic characteristics of the study population; according to NHS 2009–10 (*n* = 5293)

Age (years)	46 (31–60)
Age (age groups)	
15–24	803 (15.2)
25–44	1734 (32.8)
45–64	1743 (32.9)
> 65	1013 (19.1)
Sex	
Female	3143 (59.4)
Male	2150 (40.6)
Years of Study	10 (7–12)
Educational Level	
Low (< 8 years)	1408 (26.7)
Middle (8–12 years)	2882 (54.7)
High (≥ 12 years)	983 (18.6)
Residence area	
Rural	784 (14.8)
Urban	4507 (85.2)
MSS independent of its intensity	
No	3068 (58.8)
Yes	2148 (41.2)
MSS with intensity more than 4	
No	3254 (62.4)
Yes	1964 (37.6)

Data presented in median (P25-P75) and n (%) as appropriate

*NHS* National Health Survey; *MSS* Musculoskeletal symptoms

The HRQoL presented an average of 48.9 (95% CI 48.5–49.3) and 49.5 (95% CI 49.1–50.0) points in their physical and mental dimensions, respectively. For the physical health dimension, the subject prevalence with “low HRQoL” was 28.9% (95% CI 27.0–30.9); and 29.3% (95% CI 27.3–31.5) for the mental health dimension.

The observed population prevalence of people without MSS was 45.4% (95% CI 43.1–47.7), with aMSS was 11.9% (95% CI 19.3–13.7) and with cMSS was 42.6% (95% CI 40.4–44.9).

The HRQoL score values in their PCS and MCS showed statistically significant differences in the three MSS groups (*p* < 0.001). The scores of PCS and MCS were lower in cMSS versus aMSS, and the scores of both dimensions were lower in the two groups mentioned above when compared with those without MSS. Additionally, findings showed that in subjects with cMSS, the prevalence of “Low HRQoL” in its physical dimension was 50.5% and in the mental dimension was 39.7%. (Table 2).

**Table 2:** HRQoL scores in MSS groups, according to NHS 2009–10 (*n* = 5276)

	woMSS *n* = 2310	aMSS *n* = 562	cMSS *n* = 2404	*p*-value
PCS (physical composite score)	51.4 + 7.5	48.7 + 8.8	43.8 + 10.1	< 0.001
“High HRQoL” in PCS	1893 (81.9)	391 (69.6)	1190 (49.5)	< 0.001
“Low HRQoL” in PCS	417 (18.1)	171 (30.4)	1214 (50.5)	< 0.001
MCS (mental composite score)	51.5 + 9.1	49.7 + 9.9	47.7 + 10.6	< 0.001
“High HRQoL” in MCS	1803 (78.1)	395 (70.3)	1450 (60.3)	< 0.001
“Low HRQoL” in MCS	507 (21.9)	167 (29.7)	954 (39.7)	< 0.001

Health related quality of life, in physical and mental composite scores, in population without musculoskeletal symptoms (woMSS), with acute musculoskeletal symptoms (aMSS) and chronic musculoskeletal symptoms (cMSS)

Data presented on mean + standard deviation, and n (%), as appropriate

*HRQoL* Health related quality of life; *MSS* Musculoskeletal symptoms; *NHS* National Health Survey; *PCS* Physical composite score; *MCS* Mental composite score

When comparing HRQoL scores in people with cMSS according to control variables, the prevalence of “low HRQoL” in their physical health dimension in women (53.7%) and men (44.5%) stands out. Conversely women (46.2%) differ from their “low HRQoL” from men (27.4%) in the mental health dimension. The prevalence of “low HRQoL” in its physical dimension exceeded 50% in people over 44 years of age. Moreover, 70.4% of people with high educational demonstrated “high HRQoL” in the physical health dimension, while 70.2% of the population with chronic musculoskeletal symptoms with low educational level exhibited “low HRQoL”. Finally, 62.8% of the population from a rural residence presented with a “low HRQoL” in its physical health dimension. (See Supplementary Table 1, Additional File 1).

The results showed that the HRQoL in its physical composite score demonstrated an inverse relationship with age in the population woMSS (rho − 0.32), aMSS (rho − 0.39) and cMSS (rho − 0.39); and a direct relationship with the years of study in the population woMSS (rho 0.27), aMSS (0.34), and cMSS (rho 0.35), where all relationships were statistically significant (value *p* < 0.05). The mental composite score analysis in its relationship with age was developed in the population woMSS (rho 0.03), aMSS (rho 0.0034) and cMSS (rho 0.0202); and with the years of study in the population woMSS (rho 0.08), aMSS (rho 0.06), and cMSS (rho 0.08). This results had low correlation values (< 0.04) that were not statistically significant.

The univariate logistic regression model performed to explain the low HRQoL in its physical and mental composite scores yielded significant values for both cMSS and control variables (Table 3).

**Table 3:** Univariate logistic regression analysis, according to NHS 2009–10. (*n* = 5276)

	“Low HRQoL”			“Low HRQoL”		
	PCS			MCS		
	OR	95% CI	*p*-value	OR	95% CI	*p*-value
cMSS	3.9	3.5–4.4	< 0.001	2.1	1.9–2.4	< 0.001
Female	1.6	1.4–1.8	< 0.001	2.3	2.0–2.6	< 0.001
Age range 15–24	1	ref		1	ref	
Age range 25–44	1.9	1.5–2.4	< 0.001	1.2	1.0–1.5	=0.016
Age range 45–64	4.3	3.4–5.4	< 0.001	1.5	1.2–1.8	< 0.001
Age range > 65	10.3	8.1–13.1	< 0.001	1.4	1.1–1.7	< 0.001
High Educational Level	1	ref		1	ref	
Middle Educational Level	0.2	0.2–0.3	< 0.001	0.6	0.6–0.7	< 0.001
Low Educational Level	0.1	0.1–0.1	< 0.001	0.4	0.3–0.5	< 0.001
Urban Area	1	ref		1	ref	
Rural Area	1.8	1.6–2.1	< 0.001	1.1	1.0–1.4	=0.007

Univariate logistic regression analysis to explain the low health related quality of life in its physical and mental composite score, according to chronic musculoskeletal symptoms and control variables,

*NHS* National Health Survey; *HRQoL* Health related quality of life; *PCS* Physical composite score; *MCS* Mental composite score; *cMSS* Chronic musculoskeletal symptoms; 95% CI 95% confidence intervals, *cMSS* Chronic musculoskeletal symptoms; *OR* odds ratio; *Ref* Corresponds to the reference level on which the analysis was performed

According to the multivariate logistic regression model, the presence of cMSS as an explanation for the low HRQoL in its physical composite score, yielded an OR 3.1 (95% CI 2.7–3.5), as well as an OR 1.9 (95% CI 1.6–2.1) in its mental composite score independent of the control variables (sex, age, educational level and area of residence). In the mental composite score arena, low correlation scores and a lack of statistical significance were observed in the variables age in ranges and area of residence. Therefore, the cMSS is constituted as a statistically significant risk factor to have a low HRQoL. (Table 4).

**Table 4:** Multivariate logistic regression analysis, according to NHS 2009–10. (*n* = 5276)

	“Low HRQoL”			“Low HRQoL”		
	PCS			MCS		
	OR	95% CI	*p* - value	OR	95% CI	*p*-value
cMSS ^a^	3.1	2.7–3.5	< 0.001	1.9	1.6–2.1	< 0.001
Female (compared to male)	1.5	1.3–1.7	< 0.001	2.2	1.9–2.5	< 0.001
Age range 15–24	1	ref		1	ref	
Age range 25–44	1.6	1.2–2.0	< 0.001	1.1	0.9–1.3	=0.337
Age range 45–64	2.6	2.0–3.3	< 0.001	1.0	0.8–1.3	=0.407
Age range > 65	4.8	3.7–6.3	< 0.001	0.8	0.6–1.0	=0.136
High Educational Level	1	ref		1	ref	
Middle Educational Level	0.4	0.4–0.5	< 0.001	0.7	0.6–0.8	< 0.001
Low Educational Level	0.3	0.2–0.3	< 0.001	0.4	0.3–0.5	< 0.001
Urban Area	1	ref		1	ref	
Rural Area	1.3	1.1–1.5	=0.003	1	0.8–1.1	=0.951

Multivariate logistic regression analysis to explain the low health related quality of life in its physical and mental composite score, according to chronic musculoskeletal symptoms and control variables

*NHS* National Health Survey; *HRQoL* Health related quality of life; *PCS* Physical composite score; *MCS* Mental composite score; *cMSS* Chronic musculoskeletal symptoms; *95% C*I 95% confidence intervals, *cMSS* Chronic musculoskeletal symptoms; *OR* Odds ratio; *Ref* Corresponds to the reference level on which the analysis was performed

Odds ratio adjusted for control variables (sex, age, educational level and residence area)

^a^ Compared with the groups without cMSS (without MSS and acute MSS)

## Discussion

This study clarifies the level of involvement for HRQoL in people with cMSS, compared to those with aMSS and those with woMSS. This study found that the following are risk factors for low HRQoL in its physical dimension: the female sex, age over 44 years and rural area of residence. On the other hand, high educational level was found to be a protective factor for low HRQoL in its physical and mental dimensions.

In relation to chronic pain, cMSS worldwide prevalence demonstrate wide ranges (12 to 41%) [14], which would be explained by the different definitions for chronic pain, types of studies, data collection methodology and measurement instruments. The present study reports a higher prevalence than the upper limit of the previously reported range. The foregoing could be explained as similar prevalence is presented in studies with the same cut-off points of chronic pain (3 months duration) and similar methodologies of data collection (population surveys type face-to-face interviews) [15]. In Colombia, a cross-sectional descriptive population study in urban areas reported a chronic pain prevalence of 33.9% [7] and in Brazil, a cross-sectional population study in São Luís exhibited a 42.3% prevalence [8]. The present study results resemble similar outcomes across Latin America. A study conducted in Ireland obtained a 62.6% cMSS prevalence, which is greater than that found in the present study. This could be explained because the population from which the data were obtained, which was a sample drawn from current pain patients [16]. Subjects who were interviewed through a national telephone survey generated a low response rate (16.6%) [16].

Another factor that could create disparity in reported chronic pain prevalence centers on the high heterogeneity in chronic pain definitions and the different methodologies used for evaluating population studies worldwide. These disparities make difficult the ability to relate global epidemiological chronic pain findings with consistency in healthcare policy across countries [17, 18].

Regardless of the differences with other countries, for Chile the prevalence of cMSS is greater than the national prevalence of dyslipidemia (38.5%), hypertension (26.9%), respiratory symptoms (24.5%), depressive symptoms (17.2%) and type 2 diabetes (9.4%) [12].

The low HRQoL scores found both in mental (47.7 + 10.6) and physical (43.8 + 10.1) dimensions in cMSS Chilean population are consistent with research assessing HRQoL measured through SF-12, which suggests that quality of life is reduced in chronic pain sufferers, even when cMSS intensity is low [15]. A cross-sectional survey study developed in Japan, showed that when using SF-12 to measure HRQoL in cMSS patients and comparing those data with asymptomatic individuals, both physical (PCS 44.23 vs 47.48; *p* < 0.05) and mental (MCS 44.26 vs 51.14, *p* < 0.05) scores demonstrated differences that exceeded the established clinically relevant cut-off points, emphasizing the dramatic effect of chronic pain in the patient’s health experience [16].

In a study carried out in Brazil, people with chronic pain presented with significantly lower (*P* < 0.001) health-related quality of life scores (measured through EuroQol) [19]. In Ireland, chronic pain patients reported lower physical and mental HRQoL scores compared to the normal population [20]. The mental composite scores (MCS) were lower versus physical composite scores, which confirms that the HRQoL should be treated as a multidimensional construct [2].

The multiple logistic regression analysis shows that the cMSS variable is independent of the control variables (sex, age, educational level and ​​residence area) in its ability to explain the presence of “low HRQoL”, both in PCS as in MCS. However, this study’s findings show that the female sex is a risk factor and that the high educational level is protective factor of presenting “low HRQoL”. Increasing age seems to be a risk factor only for low HRQoL in the physical dimension. These considerations should be incorporated into national health program planning, especially in the following groups: women, elderly and people with medium and low educational level.

In relation to the approach in groups of medium and low educational levels, it is necessary to establish dissemination strategies to health professionals in primary health care, especially in vulnerable sectors. The aim of investigating subjects that, considering their educational level, could be considered more chronic in MSS added to a social context of minimum health priorities in many unsolved cases. This could be due to greater physical labor demand, ignorance of the need for early management and consultation in case of musculoskeletal diseases.

The research results illustrate the evolution of HRQoL results in MSS between the National Health Surveys 2009–2010 and NHS 2016–2017, whose data will soon be available. In Chile, the best approach to MSS management has not been determined [18]. Before concluding the best approach, biomedical and biopsychosocial factors must be further considered. The biopsychosocial approach emphasizes the patient’s self-management and their HRQoL. This explains the existing training gap that health professionals face within this epidemiological problem. This study’s findings support the need to establish standardized management policies and practices for treating chronic MSS [18].

This study was strengthened by performing the analysis on population data, which allows a generalized application and reflects Chile’s state of health. The limitations of the study center on the study methodology. Since the study used a cross-sectional analytical approach, the methodology implies some biases, such as the response bias that the subjects might have during the survey process reporting history of musculoskeletal symptoms or HRQoL. Another limitation related to the cross-sectional design of the study is that it does not imply a cause-effect relationship.

## Conclusion

Based on data from the 2009–10 National Health Survey, the scope and impact of chronic pain on the quality of life related to health in the Chilean population could be determined, and a cMSS prevalence of 42.6% (95% CI 40.4–44.9) was found.

Physical and mental dimensions in HRQoL are affected in Chilean population with cMSS, low educational level and advanced age. This is especially seen in women. This information will facilitate assessment and treatment of cMSS as a prevalent and multidimensional health problem.

## Supplementary information

**Additional file 1: Supplementary Table 1**. HRQoL in population with cMSS, according to NHS 2009–10. (*n* = 2404).

## Data Availability

The dataset analysed during the present study is available from Zenodo for researchers who meet the criteria for access doi: 10.5281/zenodo.3530819

## Abbreviations

HRQoL: Health related quality of life
cMSS: Chronic musculoskeletal symptoms
SF-12: Short form (12) health survey
NHS: National health survey
IQR: Interquartile range
OR: Odds ratio
CI: Confidence interval
IASP: International association for the study of pain
MSD: Musculoskeletal disorders
CCQ-ILAR: Community oriented program for the control of rheumatic disease (COPCORD) core questionnaire (CCQ) international league against rheumatism
MSS: Musculoskeletal symptoms
woMSS: Without musculoskeletal symptoms
aMSS: Acute musculoskeletal symptoms
SF-36: Short form (36) health survey
PCS: Physical composite scores
MCS: Mental composite scores
MSS1: Musculoskeletal symptoms of non-traumatic origin in the last 7 days, independent of the intensity of pain
MSS2: Musculoskeletal symptoms of non-traumatic origin in the last 7 days with intensity greater than or equal to 4 (of a maximum of 10) on the verbal scale of pain
Ref: Reference value
MAS: Mas adultos mayores autovalentes
