# Supplementary material for: Quality of life in chronic musculoskeletal symptomatic Chilean population: secondary analysis of National Health Survey 2009–2010
Source: BMC Musculoskelet Disord. 2020 Apr 21;21:262. doi: 10.1186/s12891-020-03261-x (PMC7175522; doi:10.1186/s12891-020-03261-x)
Supplement: Supplementary file 1 — Additional file 1: Supplementary Table 1. HRQoL in population with cMSS, according to NHS 2009–10. (n = 2404). [file 12891_2020_3261_MOESM1_ESM.docx]

| **Table 1: HRQoL** in population with cMSS**, according to NHS 2 009-10. (n= 2 404)** | | | | | | | |
| --- | --- | --- | --- | --- | --- | --- | --- |
|  | | PCS | “High HRQoL”  PCS | “Low HRQoL” PCS | MCS | “High HRQoL” MCS | “Low HRQoL” MCS |
| Sex | Male | 45.3 + 9.8 | 462 (55.5) | 370 (44.5) | 50.6 + 9.6 | 604 (72.6) | 228 (27.4) |
|  | Female | 43.1 + 10.1 | 728 (46.3) | 844 (53.7) | 46.2 + 10.8 | 846 (53.8) | 726 (46.2) |
| Age | 15-24 | 50.2 + 7.7 | 149 (73.8) | 53 (26.2) | 48.1 + 9.8 | 136 (67.3) | 66 (32.7) |
|  | 25-44 | 47.4 + 8.8 | 426 (63.4) | 246 (36.6) | 47.3 + 10,5 | 402 (5.8) | 270 (40.2) |
|  | 45-64 | 43.2 + 9.7 | 454 (48) | 492 (52) | 47.2 + 10.7 | 548 (57.9) | 398 (42.1) |
|  | > 65 | 38.8 + 9.9 | 161 (27.6) | 423 (72.4) | 48.8 + 10.6 | 364 (62.3) | 220 (37.7) |
| Educational level | High | 48.1 + 9.3 | 238 (70.4) | 100 (29.6) | 49.6 + 10.6 | 237 (70.1) | 101 (29.9) |
|  | Middle | 45.7 + 9.4 | 711 (56.7) | 542 (43.3) | 47.6 + 10.6 | 760 (60.7) | 493 (39.3) |
|  | Low | 39.4 + 9.6 | 241 (29.8) | 568 (70.2) | 47.1 + 10.5 | 451 (55.7) | 358 (44.3) |
| Residence area | Urban | 44.4 + 10.1 | 1 030 (52.2) | 942 (47.8) | 47.7 + 10.7 | 1 200 (60.9) | 772 (39.1) |
|  |  |  |  |  |  |  |  |
|  | Rural | 41.5 + 9.3 | 160 (37.2) | 270 (62.8) | 47.4 + 10 | 248 (57.7) | 182 (42.3) |
| Comparison of health related quality of life, in its physical and mental composite scores, according to control variables.  Data presented on mean + standard deviation and frequency (%), as appropriate.  HRQoL: health related quality of life; cMSS: chronic musculoskeletal symptoms; NHS: National Health Survey; PCS: physical composite score; MCS: mental composite score | | | | | | | |
